# Factors affecting the acceptance of tele-psychiatry: a scoping study

**DOI:** 10.1186/s13690-023-01146-8

**Published:** 2023-07-13

**Authors:** Reyhane Izadi, Mohammad Amin Bahrami, Mohsen Khosravi, Sajad Delavari

**Affiliations:** 1 Department of Health Care Management, School of Management and Information Sciences, Shiraz University of Medical Sciences, Shiraz, Iran; 2 Health Human Resources Research Center, School of health management and information sciences, Shiraz University of Medical Sciences, Shiraz, Iran

**Keywords:** Tele-psychiatry, Tele-medicine, Mental disorders, Mental Illness, Acceptance, Adoption

## Abstract

**Background:**

In today’s digital world, providing services through telemedicine has become an essential issue in health systems, and the Covid-19 pandemic has made this necessity even more apparent. On the other hand, mental health services are needed more than ever, and their nature makes their delivery via telemedicine more feasible than other specialized services. This study aimed to determine the factors affecting the acceptance of telemedicine among users of this technology in the field of mental health.

**Methods:**

This article is a scoping review based on the PRISMA guidelines and without any time limit until June 20, 2022. The search was performed in PubMed, Scopus, Web of Science, and PsycINFO databases using keywords related to the three fields of telemedicine, acceptance, and mental disorders. Two authors independently selected the studies based on inclusion and exclusion criteria. Then the data were collected using a data extraction form, and finally, the results were determined using the content analysis method.

**Results:**

Five main factors affect the acceptance of telemedicine among users of this technology in the field of mental health: perceived effectiveness, users’ understanding of the effects of telemedicine on the quality and outcomes of care delivery, technological aspects, organizational change capacity, the nature of the disease and psychological and psychosocial factors. These main factors are associated with 21 related sub-factors.

**Conclusions:**

Revealing the factors affecting the acceptance of telemedicine among recipients and providers of services, as key actors in health systems, can help managers and policymakers to successfully implement telemedicine in the less-regarded field of mental health, especially in the early stages.

**Supplementary Information:**

The online version contains supplementary material available at 10.1186/s13690-023-01146-8.

## Background

The digital revolution is advancing at an unstoppable pace. Along with the explosion of the digital world, mental health care is under more pressure than at any other time in history [1]. Mental disorders are a growing public health concern, and it has been estimated that depression and anxiety alone cost the global economy 1 trillion dollars in lost productivity annually [2]. Although people with mental disorders worldwide have limited access to psychological help, technological innovations are seen as a way to address the mental health crisis [1].

One of these technological innovations is telemedicine, which is used to improve the health and well-being of people in society [3]. The World Health Organization has defined telemedicine as “the use of electronic communications and information technologies to provide clinical services when participants are at different locations’’ [4].

The Coronavirus disease was diagnosed in December 2019, and the World Health Organization (WHO) defined its outbreak as a pandemic on March 2020 [5]. Many countries were slow to adopt telemedicine before the coronavirus pandemic, and as a result of the emergency created by Covid-19, telemedicine has grown tremendously worldwide in the first half of 2020 [6]. It is estimated that the use of telehealth has increased 78 times in the first two months of the epidemic [4].

Telemedicine can help improve the quality of health care, reduce costs, and facilitate health research [7]. It can also address issues such as long wait times, shortages of providers, and childcare responsibilities [8]. Despite all the benefits of using telemedicine, there are many barriers to its adoption, and the successful implementation of this technology depends on its acceptance. The high cost of implementation and poor reimbursement for care provided via telemedicine can lead to resistance to change and discourage the adoption of this technology. In addition, factors such as perceived usefulness, compatibility, perceived ease, self-efficacy, and subjective norms are effective in influencing the acceptance of this technology among providers [3]. In a general classification, the factors affecting the acceptance and use of telemedicine are divided into two levels: individual (e.g. low digital literacy) and structural (e.g. geographical location, access to broadband Internet) [8].

Despite the high prevalence of mental disorders, specialized mental health services are very limited, and this is known as the Mental Health Gap. Moreover, these services are concentrated in urban areas and this issue results in the rural-urban gap for these services. Although the expansion of human resources and infrastructure, and the integration of mental health services in primary care, seem to be two main solutions, both are associated with logistical difficulties and their implementation requires a long time. In this situation, tele-psychiatry is the only promising solution [9].

Since psychiatry is mainly based on talking to and looking at the patient, and both of these can be done through telepsychiatry, this service delivery model seems appropriate for this specialty [10]. On the other hand, evidence has shown that rural residents, particularly youth, and those who face barriers to accessing mental health counseling, are most interested in using online counseling to receive mental health services [11].

The evidence related to the factors affecting the adoption of telemedicine in mental disorders is still unclear. Studies have indicated the inherent potential of the specialized field of psychiatry to provide services through telemedicine, so determining these factors is important. This study aims to provide a scoping review to clarify the factors affecting the acceptance of tele-psychiatry in society.

## Methods

This study followed the Joanna Briggs framework [12] to conduct a scoping review. Scoping reviews can determine the main components and related aspects of a specific concept, and thus, help to draw a thematic map based on the collected evidence and identify the knowledge gaps in the scope [13]. In this research, we carried out the following five steps:

### Identification of the research question

The research question was: What are the main factors influencing the acceptance of tele-psychiatry?

PPC (Population, Content, and Context) was defined for the scoping review in the first step. The population included all countries, and with regard to the content, all factors and elements affecting the acceptance of telemedicine in the field of mental health services were considered. Regarding the context, all the cultural, geographical, and demographic characteristics, as well as the interests and attitudes that influence the acceptance of telemedicine, were regarded.

### Searching and retrieving relevant studies

A systematic search was used to identify all original published articles related to factors affecting the acceptance of telemedicine in the field of mental health, with no time limit until June 20, 2022. PubMed, Scopus, Web of Science, and PsycINFO databases were searched. The search strategy is presented in Table 1.

We used MeSH terms to categorize all keywords into three groups: acceptance, telemedicine, and mental disorder. We applied the logical operator “OR” to all synonymous keywords, and then merged the keywords of the first, second, and third groups with the logical operator “AND”. We managed references using EndNote X7.1 software. We searched with no time limits and included all original research articles that examined the acceptance of telemedicine in mental health, provided that they were in English. We excluded articles that only evaluated the use of telemedicine and did not address the factors affecting its acceptance. We also excluded review articles, short communications, letters to the editor, and other irrelevant articles from the study.

**Table 1:** Search strategy

**Timespan:** Until 2022			**Language:** English
**Strategy**: #1 AND #2 AND #3			
**Conceptual Areas**	**NO**	**Search query**	
Telemedicine	#1	“Mobile Health” OR mHealth OR m-Health OR eHealth OR e-Health OR telemedicine OR tele-medicine OR telehealth OR tele-health OR telemonitoring OR tele-monitoring OR telecare OR tele-care OR Telemental OR Tele-mental OR Telepsych* OR Tele-psych* OR telepsych* OR “tele psych*” OR Teletherapy OR Tele-therapy OR “Video counsel*” OR Video-counsel* OR “video counsel*” OR Telemedicine	
Acceptance	#2	accept* OR “behavioral intention” OR “intention to use” OR adoption OR “technology acceptance model*”	
Mental disorders	#3	“Mental Disorders” OR “Mental Disorder” OR “Psychiatric Illness” OR “Psychiatric Illnesses” OR “Psychiatric Diseases” OR “Psychiatric Disease” OR “Mental Illness” OR “Mental Illnesses” OR “Psychiatric Disorders” OR “Psychiatric Disorder” OR “Behavior Disorders” OR “Behavior Disorder” OR “Psychiatric Diagnosis” OR “Mental Disorders” OR “mood disorder” OR “psychotic disorder”	

### Inclusion of relevant studies

We retrieved articles based on the Preferred Reporting Items for Systematic Reviews and Meta-Analyses (PRISMA) flowchart [14]. Duplicate articles were removed and the remaining ones were screened based on the title and abstract. Irrelevant articles were excluded, and the full text of all the remaining articles was read. Those that met the eligibility criteria were included in the final analysis. Two researchers independently performed the entire process.

### Data extraction and charting and final collation

Two authors independently extracted data from the final articles that met the study objective. They used a data extraction form in Microsoft Office Excel 2013 for data charting. This form included sections such as authors, title, year of publication, place of research, study purpose, study design and data collection method, type of technology/platform, study purpose, target population, type of mental disorder, and factors affecting the acceptance of telemedicine. The data extraction and charting were continuous, and the charted data were analyzed using the thematic analysis approach [15].

### Summarizing and reporting the results

The data extracted and charted from the previous step were analyzed using qualitative thematic analysis and an inductive approach [16]. The authors read all the included articles and extractions several times to familiarize themselves with the data and then identified the initial codes of each meaningful extraction. All initial codes were reviewed and finalized before sub-themes (sub-factors) and main themes (main factors) were categorized, labeled, and tabulated. A map of the relationships between sub-factors and main factors was also presented to better understand the concept and achieve the aim of the scoping review.

## Results

**Fig. 1:**
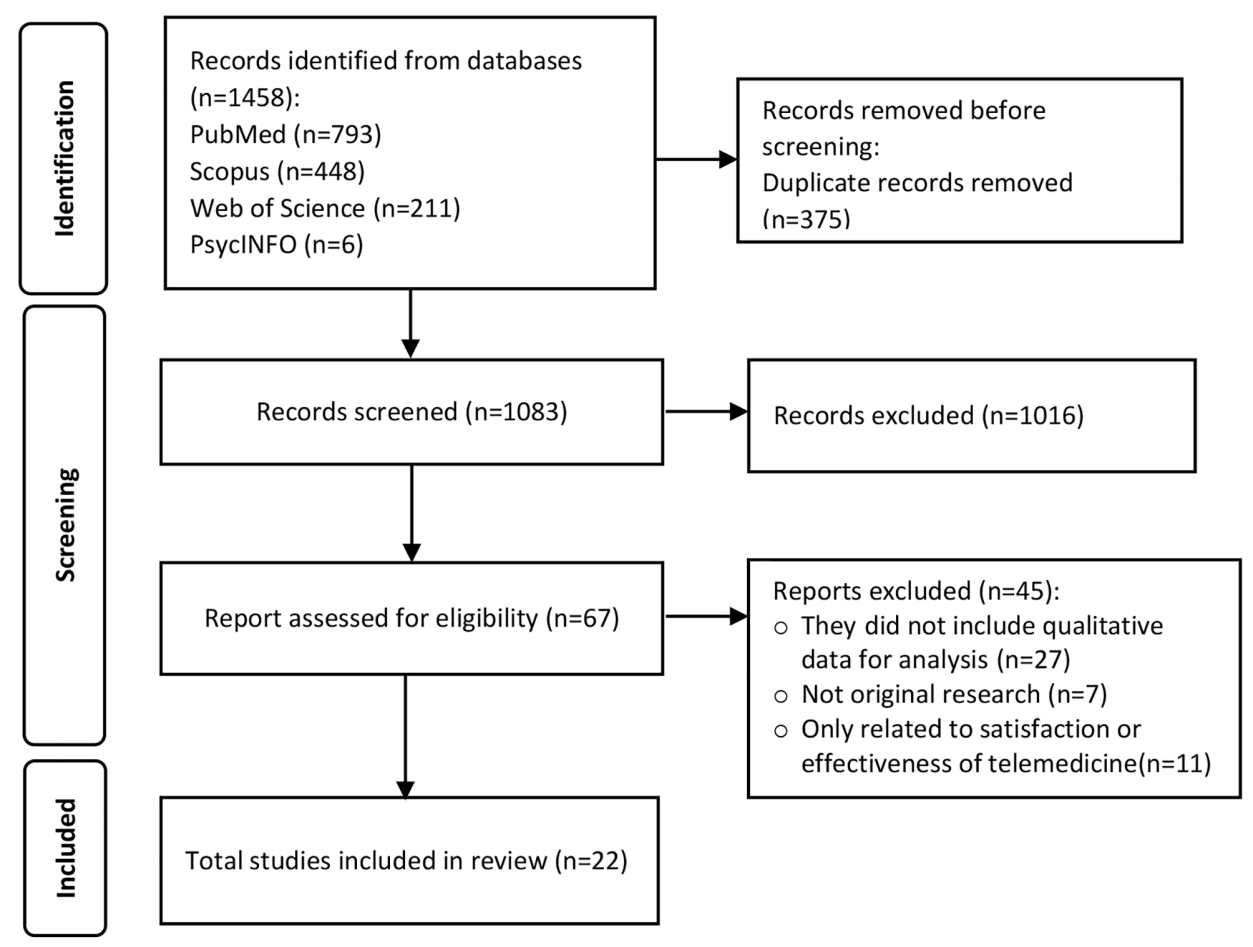
PRISMA flow chart for study selection

For this review, we searched databases and retrieved 1458 articles, of which we removed 375 duplicate studies and excluded 1016 articles based on title and abstract screening. We then reviewed the full text of 67 studies and included 22 articles in the analysis (Fig. 1).

From a total of 22 studies extracted for analysis, 12 studies identified the factors affecting the acceptance of telemedicine among service recipients from the perspective of the recipients themselves. Five studies examined these acceptance factors among providers and from the perspective of the providers themselves, and finally, five studies extracted these factors in general among recipients and providers by examining the viewpoints of both groups (other characteristics of the selected studies are presented in “Additional file 1” (Table A)).

From a total of 22 studies ultimately selected for analysis, five main factors and 21 sub-factors affecting the acceptance of telemedicine among users of this technology in the field of mental health were identified (see Additional file 2 (Table B)). The main factors were perceived effectiveness, users’ understanding of the effects of telemedicine on the quality and outcomes of care delivery, technological aspects, organizational change capacity, the nature of the disease and psychological and psychosocial factors (Table 2), each of which is described in detail below:

### Perceived effectiveness

One of the main factors identified in the selected studies is the issue of perceived effectiveness, and 20 (90.90%) studies emphasized its importance. This main factor includes two sub-factors: denial of efficacy or belief in usefulness to improving health that affects both provider and recipient acceptance, and expected financial advantages of telemedicine that only affects provider acceptance.

### Users’ understanding of the effects of telemedicine on the quality and outcomes of care delivery

Another main factor, which is identified in the current study and emphasized in 13 (59.09%) articles, is users’ understanding of the effects of telemedicine on the quality and outcomes of care delivery. It includes four sub-factors, one of which is the effect of telemedicine on the quantity and quality of relationships that affect the acceptance of recipients and providers. Telemedicine as a clinical decision support tool is another sub-factor that only affects providers’ acceptance. Telemedicine as a tool for disease self-management and telemedicine as a pathway for easy access to services are two other sub-factors that only affect recipient acceptance.

### Technological aspects

The next main factor is technological aspects, which was mentioned in 17 studies (27.77%). This main factor includes five sub-factors: platform design based on aesthetic principles, personalization, credibility, privacy, and digital literacy and ease of learning and using telemedicine, all of which influence the acceptance of this technology by both the service provider and recipient.

### Organizational change capacity

Another main factor affecting the acceptance of telemedicine among providers is the organizational change capacity, and it was mentioned in 8 articles (36.36%). Two sub-factors of this main factor are organizational-infrastructural challenges, and organizational structure and capabilities.

### The nature of the disease and psychological and psychosocial factors

Another main factor affecting the acceptance of telemedicine is the nature of the disease and psychological and psychosocial factors. This main factor is mentioned in 17 studies (77.27%) and includes eight sub-factors. Three sub-factors, including the type of disorder, social influence, and desire for a technology-based lifestyle, affect the acceptance of the service recipient and service provider. And five sub-factors, including autonomy, embarrassment and fear of mental disorder stigma in society, hedonic motivation, ease of self-disclosure, and feeling familiar due to previous experiences, only affect the recipient’s acceptance.

**Table 2:** Factors affecting the acceptance of tele-psychiatry

Main-Factors	Type of user	Sub-Factors
Perceived effectiveness	Provider & Recipient	Denial of efficiency or belief in usefulness for improving health
	Provider	Expected financial advantages of telemedicine
Users’ understanding of the effects of telemedicine on the quality and outcomes of care delivery	Provider & Recipient	The effect of telemedicine on the quantity and quality of relationships
	Provider	Telemedicine as a clinical decision support tool
	Recipient	Telemedicine as a tool for disease self-management
	Recipient	Telemedicine as a pathway to easy access to services
Technological aspects	Provider & Recipient	Platform design based on aesthetic principles
	Provider & Recipient	Personalization
	Provider & Recipient	Credibility
	Provider & Recipient	Privacy
	Provider & Recipient	Digital literacy and ease of learning and using telemedicine
Organizational change capacity	Provider	Organizational structure and capabilities
	Provider	Organizational - infrastructural challenges
The nature of the disease and psychological and psychosocial factors	Provider & Recipient	Type of disorder
	Recipient	Autonomy
	Recipient	Embarrassment and fear of mental disorder stigma in society
	Recipient	Hedonic motivation
	Provider & Recipient	Social influence
	Provider & Recipient	Desire for a technology-based lifestyle
	Recipient	Ease of self-disclosure
	Recipient	Feeling familiar due to previous experiences

**Fig. 2:**
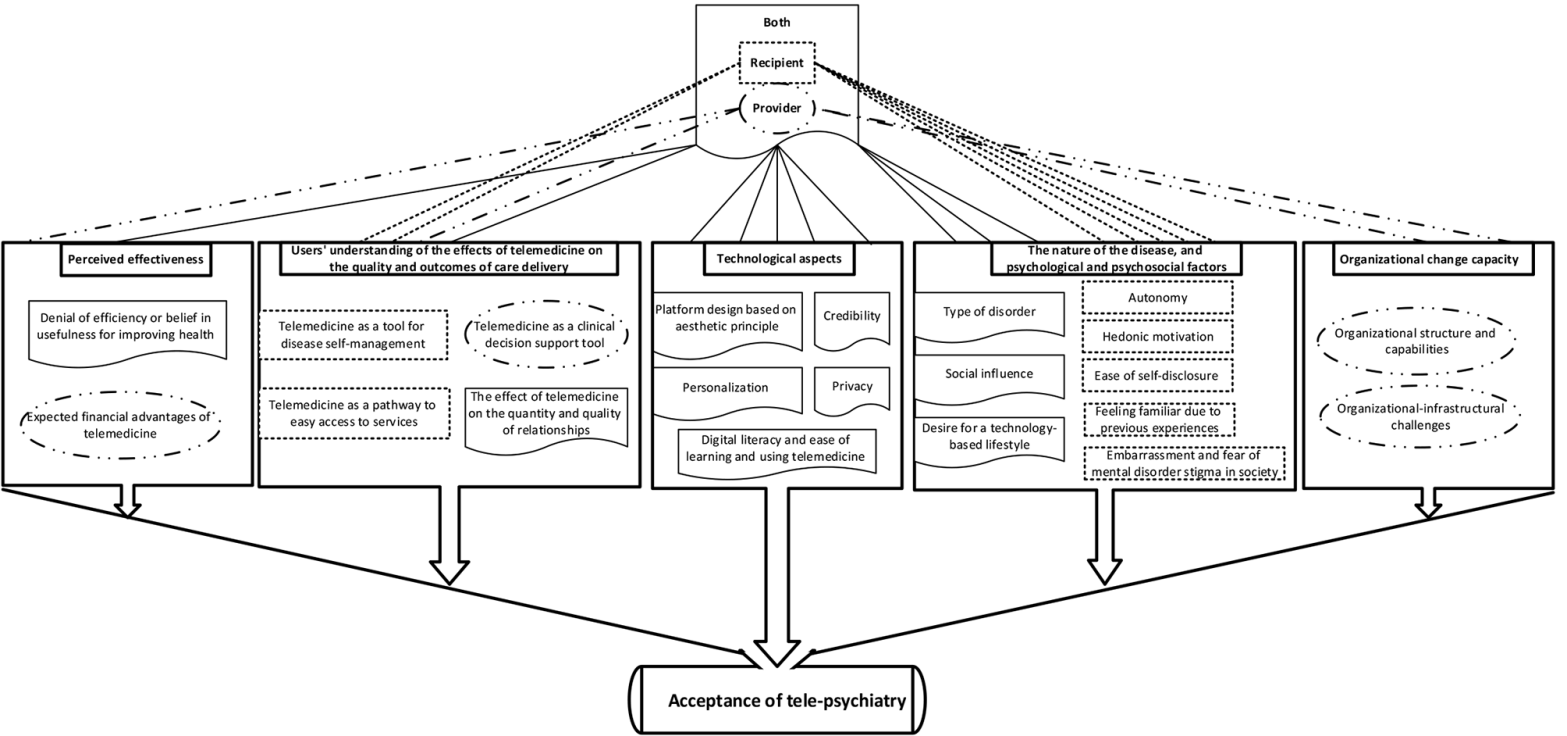
Thematic map of factors affecting the acceptance of tele-psychiatry

Finally, Fig. 2 shows the possible relationships between the main factors that can affect the acceptance of tele-psychiatry. In total, five main factors and 21 sub-factors were identified. Four sub-factors only affect the acceptance of service providers and seven sub-factors only affect the acceptance of service recipients, and 10 sub-factors affect the acceptance of this technology in both groups.

Users’ understanding of the effectiveness of telemedicine affects its acceptance. Acceptance of telepsychiatry is likely to increase if patients and providers generally have positive beliefs about its ultimate effectiveness in improving health, and if providers are optimistic about the potential financial advantages it will provide. In addition, telemedicine affects the quality and outcomes in the service delivery process, and users’ understanding of them can lead to the acceptance and rejection of this technology. For example, some service recipients consider telemedicine as a tool for self-management of the disease and easy access to services, while providers see it as a support tool for better decision-making, and these positive attitudes strongly affect the acceptance of telemedicine. Also, telemedicine affects the quantity and quality of relationships. If users perceive the communication space created by telemedicine as an opportunity for more interactions, they are more likely to accept it; but if they see it as a factor leading to a decrease in the quality of relationships, they may reject it. Another main factor is technological aspects. Compliance with the principles of credibility and privacy, as well as a good design that provides aesthetic criteria and personalization options, and enables easy use of the telemedicine platform, all facilitate the adoption of this technology among all types of users. Another main factor affecting the acceptance of this technology among the organizational providers is the organizational change capacity. Provider organizations should be able to create a reasonable balance between challenges and capabilities in the telemedicine implementation process.

Since it is not possible to provide virtual services for some disorders due to their nature, the type of mental disorder affects the decision to accept this technology among all users. Also, some psychosocial factors) for example, embarrassment and fear of mental disorder stigma in society) and some psychological factors (for example, autonomy) have an effect on the decision to accept this technology.

## Discussion

The aim of the study was to determine the factors affecting the acceptance of telemedicine among the users of this technology in the field of mental health. Five main factors and 21 sub-factors were identified, each of which is discussed in detail below:

### Perceived effectiveness

The main factor of *perceived effectiveness* includes two sub-factors, which are related to the final impact of telemedicine on health status and financial advantages. One of the sub-factors identified among providers and recipients of health services is *denial of efficiency or belief in usefulness for improving health*, which reflects conflicting perspectives. While some consider telemedicine useful and effective for improving health, others prefer other service models and do not believe in the effectiveness of this technology. Garavand et al. introduced perceived usefulness as an important factor affecting the acceptance of telemedicine among physicians [3]. Ramírez-Correa et al., by determining the behavioral theory and telemedicine acceptance model among patients during the COVID-19 pandemic, showed that perceived usefulness effectively influences behavioral intention [17]. The next sub-factor is the *expected financial advantages of telemedicine* for the providers. The providers’ willingness to accept telemedicine increases with their positive prediction of the possible financial benefits from its implementation. Evidence has also shown that digital health interventions are an attractive option for reducing staff and costs in organizations, and therefore organizations are encouraged to use this technology [18, 19].

### Users’ understanding of the effects of telemedicine on the quality and outcomes of care delivery

The next main factor is *users’ understanding of the effects of telemedicine on the quality and outcomes of care delivery*, which includes four sub-factors. The first sub-factor is *the effect of telemedicine on the quantity and quality of relationships*. Some users consider telemedicine as a relationship facilitator that provides an opportunity for more interactions and socialization [20, 21]. Similarly, one study found that telemedicine provides more opportunities for communication between patients and other people involved in their health [22]. Another study showed that the duration of video counseling is almost half of the duration of face-to-face counseling; this was a motivating factor for the acceptance of telemedicine among psychotherapists because they could provide more visits via telemedicine than in-person services [19]. Some other users believe that telemedicine faces communication challenges and hinders relationships due to the lack of face-to-face contact, non-verbal and personal interaction. In this regard, evidence has shown that the quality of relationships is strongly influenced by visual and non-verbal communication. It has also been found that patients often misunderstand health information provided over the phone [23]. Another study mentioned that specialist physicians are hesitant to use this technology because they believe that their ability to communicate with the patient in face-to-face consultation is higher than in virtual consultation; this is due to the lack of objective information in virtual visits, whereas a physician usually obtains this information during a traditional consultation [24]. The next sub-factor is *telemedicine as a clinical decision support tool*. Telemedicine strengthens professional interactions, and many providers see it as a tool for professional team building and knowledge sharing to make the best decisions [25]. Similarly, others have shown that telemedicine can facilitate communication between professionals, provide a context for increased peer support, and ultimately lead to more accurate decision-making. These factors increase the willingness to adopt it among the providers [25, 26]. *Telemedicine as a tool for disease self-management* is another sub-factor. Pugliese and Wolff stated that telemedicine as a tool for self-management shifts the control from the therapist to the patient, enhances a sense of active responsibility, and leads to the completion of individual therapeutic tasks and goals [27]. Others have introduced telemedicine as a way to increase patient self-sufficiency [28] and a powerful tool to stimulate thought in self-management of severe mental health problems [18]. The last sub-factor is *telemedicine as a pathway to easy access to services*. Indeed, the use of telemedicine results in a reduction in waiting time, flexibility in planning, and the elimination of the need to spend time and money on travel, and as a result, access to services is facilitated. Barbosa et al. stated that telemedicine ideally leads to improved access by solving the problems of transportation and geographic distance [26]. Pang et al. presented ease of access to care as one of the factors that influence the acceptance of telemedicine in elderly cancer patients [29].

### Technological aspects

The next main factor is *technological aspects*. This main factor includes five sub-factors, all of which affect the acceptance of both providers and recipients of health services. *Platform design based on aesthetic principles* is the first sub-factor that refers to the impact of the appearance of the platform on the acceptance of this technology. Other researchers have also introduced some of these appearance features, such as application format, color scheme, program graphics, and zoom quality, as factors influencing platform acceptance [28, 30, 31]. The second sub-factor is *personalization*. The design of the platform’s infrastructure is equally important as its appearance design, as it enables the adaptation of the telemedicine platform and the service delivery process to the preferences, needs, and characteristics of various users [30, 32]. A study that investigated the acceptance of telemedicine among students showed that one of the most important factors influencing behavioral intention to use is perceived personalization [33]. The next two sub-factors are *credibility* and *privacy*. The acceptance of a telemedicine platform is influenced by the presence of valid certifications, as well as the privacy concerns and the degree of immunity to hackers that it offers. Similarly, Brewster et al. found that credibility was one of the factors that influenced the acceptance of telemedicine [34]. Moreover, Hall and McGraw reported that privacy and security risks in telehealth systems could adversely affect the trust and readiness of patients and doctors to adopt and use it [35]. The last sub-factor is *digital literacy and the ease of learning and using telemedicine*. This means that the ease of use of telemedicine, which originates from digital literacy and the ease of learning the telemedicine platform, will ultimately affect the adoption of telemedicine. One study investigated the obstacles and enablers for telehealth in dementia management and found that the most significant factors influencing the acceptance of this technology among patients and providers were the ease of learning and the ease of working with telemedicine [36]. Similarly, another study explored the factors associated with the adoption and utilization of an Internet intervention by the caregivers of patients with dementia and revealed that one of these factors was the caregivers’ perception of the ease of use [37].

### Organizational change capacity

Another main factor is *organizational change capacity*, which influences the adoption of telemedicine by service provider organizations. Some intellectual concerns related to the implementation of telemedicine influence its adoption by provider organizations. This main factor is not a fixed characteristic of an organization, but rather a dynamic and contextual one that depends on the balance of capabilities and challenges [38]. Accordingly, this main factor comprises two sub-factors: *organizational structure and capabilities* and *organizational-infrastructural challenges*. The first sub-factor is *organizational structure and capabilities*. In fact, organizational culture, organizational agility level, available resources, skills and expertise within the organization are all effective in the willingness of the organization to accept this technology [19, 31]. Nyamu et al. have highlighted the role of organizational agility and resources in facilitating telemedicine adoption [39]. The second sub-factor is *organizational-infrastructural challenges*. Some concerns about the challenges that the organizations face to use telemedicine and the organizations’ prediction of how and the possibility of solving these challenges influence the providers’ willingness to take the first steps towards this technology. Internet speed is one of the most important of these types of challenges [25]. Moreover, other organizational problems such as technical challenges [40] and the challenge of providing resources to offer incentives and reasonable financial rights for providers [19], and other possible challenges that the organization faces, as well as the organization’s anticipation of possible support to overcome these challenges [41], all influence the decision of organizations to accept this technology [40].

### The nature of the disease, and psychological and psychosocial factors affecting acceptance

Since this study is about the acceptance of telemedicine in the field of mental health interventions, one of the most important main factors identified is *the nature of the disease and psychological and psychosocial factors affecting acceptance*. This factor includes eight sub-factors. One of the most important sub-factors affecting the acceptance of telemedicine in all users is the *type of mental disorder*. Determining a suitable target group that is compatible with this type of intervention is very important. Rangachari et al. showed that the type of medical specialty has an effect on the acceptance of telehealth; so that psychiatry, cardiology, and radiology specialties used this technology the most, and immunology and gastroenterology specialties used this technology the least [42]. The non-pharmacological nature of some mental health interventions is one of the main reasons for the acceptance of telemedicine in this field [21]. Hoffmann et al. stated that telemedicine is less appropriate for patients with certain mental health conditions such as post-traumatic stress disorder [19]. The next sub-factor is *autonomy*. So that the more prominent this feature is in service recipients, the more their willingness to accept telemedicine increases. Cimperman et al. showed that the level of self-efficacy of elderly patients influences their behavioral intention and that higher willingness to accept telemedicine is related to their higher level of self-efficacy [43]. Rubeis et al. stated that this type of intervention requires a certain level of autonomy and may enhance it. For example, patients with cognitive or audio-visual disorders may not be able to use this technology well [44]. The third sub-factor is the *embarrassment and fear of mental disorder stigma in society*, which strongly affects the acceptance of telemedicine among mental health service recipients. Benjat et al. stated that the feeling of ashamed related to the illness has a positive effect on the acceptance of electronic mental health services [45]. A qualitative study of users’ perspectives on digital health interventions, revealed that individuals with severe mental health disorders perceived these interventions as a desirable means of alleviating the fear of social judgment [18]. The next sub-factor is *hedonic motivation*, which refers to the fact that some service recipients find using the Internet attractive and a way to satisfy their curiosity, and consider digital health interventions as a fun method [18, 21]. The fifth sub-factor that affects the adoption of telemedicine among all types of users is *social influence*. Some people decide to use or not use Internet services under the influence of encouragement or lack of encouragement from their family, friends, colleagues, and the surrounding community in general [46, 47]. *Desire for a technology-based lifestyle* is another sub-factor that affects telemedicine adoption for both service providers and recipients. Some people have a desire to improve their lifestyle and use up-to-date technologies in all aspects of their lives, which also influences their acceptance of telemedicine [21, 37]. The seventh sub-factor is *feeling familiar due to previous experiences*, which is related to service recipients. Evidence has shown that previous familiarity with the service delivery environment [40] and the existence of a stable relationship between the patient and the therapist before starting to provide telemedicine services can affect the behavioral intention of service recipients to use telemedicine systems [19]. The last sub-factor is the *ease of self- disclosure*. In fact, people feel more comfortable in telemedicine than in face-to-face visits. Mozes et al., in their study of patients’ preferences for telemedicine versus face-to-face services, showed that patients felt more comfortable with a variety of virtual medical services [48].

### Strengths and limitations of the study

One of the main strengths of this study is a comprehensive analysis of factors affecting the acceptance of telemedicine from the perspective of both providers and recipients of services in the often-neglected field of mental health services. However, future research is recommended to conduct a systematic review on this issue and examine the factors affecting the acceptance of telemedicine among different types of users separately in more depth. Furthermore, providing a map to depict how the influencing factors are related to each other can be considered another strength of the current research. Nevertheless, the first and most important limitation of the current research is that the selected studies are only in English.

## Conclusions

This qualitative study aimed to explore the factors that influence the acceptance of telemedicine in the field of mental health. The results indicate five main factors and 21 sub-factors affecting the acceptance of tele-psychiatry. Some of these sub-factors influence the decision to accept this technology by both provider and recipient groups, while some only pertain to the decision of one of these groups. Overall, if users have a positive understanding of the ultimate effectiveness of telemedicine in improving health, and especially if the providers consider the predictions of financial advantages resulting from its implementation to be satisfactory, the tendency to accept telemedicine increases. Furthermore, the effects of telemedicine on the outcomes and quality of the service delivery process influence its acceptance. Its effects on the quantity and quality of relationships determine its rejection and acceptance. Easy access to the service and the ability to self-manage the disease are among the most important immediate outcomes of telemedicine for service recipients, and for providers, it is considered a support tool for better decision-making. The technological aspects, including the appropriate design for its appearance and infrastructure, that meet both the aesthetic criteria and the possibility of personalization, affect the acceptance of this technology. Users should be able to be sure of the credibility of the platforms and respect for their privacy in order to ultimately accept it. Additionally, their understanding of the ease of using these platforms influences their intention to use this technology. The flexibility and capacity to change the organization for the establishment of tele-psychiatry is also one of the main factors affecting the acceptance of this technology by organizational providers. Moreover, the acceptance of digital interventions in the field of mental health depends on one of the basic issues, which is the type of mental disorder and related mental factors. The treatment of some disorders involves a great complexity that requires face-to-face evaluations and physical examinations. Similarly, a person’s mental factors affect preferences, expectations, attitudes, beliefs and motivations for accepting telepsychiatry.

This research provides insights into the path for the successful implementation of telemedicine in the treatment of mental disorders, which can be used by policymakers and decisionmakers in this field. For more precise results in this field, it is suggested that these factors be determined based on population groups and by the type of mental disorder, and each of these factors should be investigated separately and more accurately.

## Electronic supplementary material

Below is the link to the electronic supplementary material.

Supplementary Material 1

Supplementary Material 2

## Data Availability

The datasets used and/or analyzed during the current study are available from the corresponding author on reasonable request.

## Abbreviations

WHO: World Health Organization
DHIs: Digital health interventions
PRISMA: Preferred Reporting Items for Systematic Reviews and Meta-Analyses
COVID-19: Coronavirus Disease 2019
CMDs: Common mental disorders
M-Health: Mobile health
App: Application
E-Mental Health Programs: Electronic Mental Health Programs
