# Supplementary material for: Factors affecting the acceptance of tele-psychiatry: a scoping study
Source: Arch Public Health. 2023 Jul 13;81:131. doi: 10.1186/s13690-023-01146-8 (PMC10339628; doi:10.1186/s13690-023-01146-8)
Supplement: Supplementary file 1 — Supplementary Material 1 [file 13690_2023_1146_MOESM1_ESM.docx]

| **Table A** Characteristics of selected studies | | | | | | | | | | | |
| --- | --- | --- | --- | --- | --- | --- | --- | --- | --- | --- | --- |
| **Item** | **Author(s)** | **Title** | **Year of publication** | **Place of research** | **Study aim** | **Study design (data collection method)** | **Type of technology /platform** | **Perspective** | **Target population** | **Type of mental disorder** | **The factor affecting the acceptance** |
| 1 | S. F. Austin, et al.[1] | Service user experiences of integrating a mobile solution into clinical treatment for psychosis | 2021 | Western Europe | Evaluation of patient experience of using M-health | Qualitative (Semi-structured interview) | M-Health | Patient | Patient | Psychosis | - Easily Accessible and Supporting Memory, Promoting Dialogue, Encouraging Reflection, Factors That Affect Engagement, |
| 2 | M. S. Bauer, et al. [2] | Implementing and Sustaining Team-Based Telecare for Bipolar Disorder: Lessons Learned from a Model-Guided, Mixed Methods Analysis | 2018 | USA | Probing the factors affecting the experience of video teleconference approach | A mixed method research (semi-structured interview & Data recorded in service provider clinics) | Video teleconference | Provider and Patient | provider and Patient | Bipolar Disorder | - Key facilitators included valued recommendations, ease of use and integration into ongoing workflow via electronic health record for consulting providers, and extensive infrastructure at the national level to support implementation, - Notable barriers included the labor-intensive nature of scheduling; variable availability of telehealth space, equipment, and staff at certain sites, - Other: Geographical region,   Activity start date, Inner and outer context**,** |
| 3 | Corina Benjet ,et al. [3] | Treatment delivery preferences associated with type of mental disorder and perceived treatment barriers among Mexican university students | 2020 | Mexico | Assessing willingness to use treatment and treatment method preferences | Quantitative  (Web based survey) | Internet based electronic health (eHealth) | Patient (Students) | Patient (Students with mental disorders) | Depression, Attention deficient hyperactivity | - Being embarrassed, Worried about harm to one's academic career, wanting to handle problems on one's own, Beliefs about treatment efficacy, |
| 4 | N. Berry, et al. [4] | A qualitative exploration of service user views about using digital health interventions for self-management in severe mental health problems | 2019 | UK | Examining service users' views of digital health for severe mental health problems | Qualitative (interview) | Digital health interventions (DHIs) | Patient (user) | Patient (with severe mental problems) | Schizophrenia, Bipolar disorder | - DHIs as powerful tools to stimulate reflection, understanding and change, - DHIs as an increaser of existing gaps due to digital divide, - Concerns about who has access to DHI data and how, - DHIs as an attractive option to reduce the number of employees, reduce costs and reduce the fear of being judged in face to face visits, - DHIs as a positive, fun, practical and interactive way of self-management, |
| 5 | C. Bleyel, et al. [5] | Patients' perspective on mental health specialist video consultations in primary care: qualitative pre implementation study of anticipated benefits and barriers | 2020 | Germany | Determining patient opinions about video counseling | Qualitative (interview) | Video Consultations | patient | Patient | Depression or Anxiety | - Anticipated Benefits: Shorter Waiting Times, Shorter Travel Distances, Lower Threshold for Seeking Specialist Mental Health Care, Familiar Primary Care Environment, - Anticipated Barriers: Lack of Face to Face Contact, Technical Challenges, Organizational Challenges, Stigma of Seeking Mental Health Care, - Prerequisites for Interacting with Providers in Video Consultations: Empathy, being taken seriously, and feeling as necessary prerequisites, |
| 6 | Y. M. Chae, et al. [6] | The reliability and acceptability of telemedicine for patients with schizophrenia in Korea | 2000 | Korea | Probing Examining patient opinions about telemedicine and face to face medicine | Qualitative (interview) | Telemedicine | patient | Patient | Schizophrenia | - Feeling comfortable, Ease of self-expression, Quality of interpersonal relationship, Usefulness, |
| 7 | T. M. Chiu and G. Eysenbach [7] | Stages of use: consideration, initiation, utilization, and outcomes of an internet mediated intervention | 2010 | Canada | Investigating factors associated with uptake and use of an Internet intervention for caregivers of patients with dementia | A mixed method research (Interview & Questionnaire) | E-mails | Provider (Caregiver) | Provider (Caregiver) | Dementia | - Usefulness, Attitude towards higher technology, Caregivers' perception of ease of use |
| 8 | N. Coley, et al. [8] | Older adults' reasons for participating in an e-health prevention trial: a cross country, mixed methods comparison | 2018 | Finland, France, Netherlands, | To explore older adults' reasons for participating in a multinational eHealth prevention trial, and compare motivations between countries | A mixed method research (Online questionnaire & semi structured interview) | E-Health | patient | Patient (Older Adults’) | Memory disorders (without dementia) | - Reasons: Considering the internet as fun, Internet platform as an effective way to improve health, convenience of continuous access, contributing to scientific progress and contributing to the development of an Internet health tool, wanting to improve one's lifestyle, benefiting from additional medical monitoring, sense of reliability and trustworthiness, as an opportunity to interact, as a way to satisfy curiosity, the non-pharmacologic nature of the intervention, - Barriers: lack of conﬁdence in computer skills, the importance of social interaction and communication, |
| 9 | V. Cristancho Lacroix, et al. [9] | A web-based psychoeducational program for informal caregivers of patients with Alzheimer's disease: a pilot randomized controlled trial | 2015 | France | Efficacy and acceptability of a Web-based psychoeducational program for informal caregivers of persons with Alzheimer’s disease (PWAD) | A mixed method research (semi structured interview & questionnaire) | Web -based fully automated psychoeducational program (called Diapason) | Provider (Caregiver) | Provider (Caregiver) | Alzheimer’s disease (PWAD) | - Maintaining autonomy, Dynamism, Flexibility, Personalization, Socialization, perceived usefulness for caregiver & patient, Preference of another service model from the patient's point of view, |
| 10 | V. P. Dal Bello Haas, et al. [10] | Lessons learned: feasibility and acceptability of a telehealth delivered exercise intervention for rural dwelling individuals with dementia and their caregivers | 2013 | Canada | Feasibility of a telehealth delivered exercise intervention for people with dementia and their caregiver | A mixed method research (questionnaire & interview) | Telehealth | Patient and Provider (caregiver) | Patient and Provider (caregiver) | Alzheimer, Dementia | - Ease of getting to the telehealth department, how well privacy was respected, Ability to focus without distraction due to telehealth, Previous experiences, |
| 11 | L. P. S. Dias, et al. [11] | Development and testing of i-Aware model for ubiquitous care of patients with symptoms of stress, anxiety and depression | 2020 | Brazil | Providing a model for comprehensive care of patients with anxiety, depression and stress disorders | Quantitative (Survey) | Gamification and biodata (i-Aware) | Patient | Patient | Anxiety, Depression, Stress | - Usefulness, Easy to understand, Usability, |
| 12 | C. Doyle, et al. [12] | Videoconferencing and tele mentoring about dementia care: evaluation of a pilot model for sharing scarce old age psychiatry resources | 2016 | Australia | Evaluation of a pilot model for expanding geriatric psychiatric consultation services | A mixed method research (semi structured interview & questionnaire) | Video conferencing,  Tele mentoring | Provider (caregiver) | Provider (caregiver) | Dementia | - Reductions in travel times, Increasing peer support and team building, Internet speed, |
| 13 | G. Fergie, et al. [13] | Social media as a space for support: Young adults' perspectives on producing and consuming user generated content about diabetes and mental health | 2016 | UK | Determinants affecting the acceptance of these technologies by users in the daily experience of health status | Qualitative (semi structured interview) | Social media | Patient (user, young adult) | Patient (user, young adult) | Diabetes or a common mental health disorder (CMHD) | - Experiences of offline support, Desirability of contributing any health-related content, Concerns about compromising their identity, Concerns about whether content is appropriate for sharing, |
| 14 | S. Fitrianie, et al. [14] | Factors affecting user's behavioral intention and use of a mobile phone delivered cognitive behavioral therapy for insomnia: a small scale analysis | 2021 | Netherlands | Explaining the difference between people's behavioral intention and their usage behavior to use an insomnia program | Quantitative (Questionnaire) | Mobile App | Patient (user) | Patient (user) | Insomnia | - Performance expectancy, Effort expectancy, Social influence, Self-efficacy, Trust, Anxiety, Facilitating conditions, Behavioral intention, |
| 15 | A. Fonseca, et al. [15] | Women's use of online resources and acceptance of e-mental health tools during the perinatal period | 2016 | Portugal | Describing the pattern and examining the determinants in the use of online resources for psychological problems in the perinatal period | Quantitative (survey) | E-health | Patient (user) | Patient (user) | Perinatal depression | - The individual’s attitudes (effective & attractive), The individual’s perceived behavioral control (perceptions of internal and external constraints), The individual’s subjective norms (To encourage or not to encourage family and friends to use), The perceived usefulness, The perceived ease of use, |
| 16 | M. E. Gately, et al. [16] | Factors influencing barriers and facilitators to in home video telehealth for dementia management | 2022 | USA | Barriers and facilitators to telehealth for dementia management | Qualitative (semi structured interviews) | Video telehealth | Provider (caregiver) | Patient and Provider (caregiver) | Dementia | - Increasing access to care, Potential to reduce the need for travel (both in terms of distance and traffic), Relative ease with video conferencing, for personal reasons, Ease of learning to use remote video health for ease of operation, Cost, Relationship with other patients in the platform, Immunity of the platform in front of hackers, |
| 17 | M. Hoffmann, et al. [17] | Perspectives of psychotherapists and psychiatrists on mental health care integration within primary care via video consultations: qualitative pre implementation study | 2020 | Germany | To explore the acceptance of video consultations embedded in primary care | Qualitative (semi structured focus groups) | Video based mental health care | Patient and Provider (Mental health specialists) | Provider (mental health specialist) | Mental health disorder | - Individual Health Professional Factors: Time and duration of the individual video consultation, The absence of personal interactions and nonverbal, Doubts about the appropriateness of video consultations to specialized mental health care, The existence of a stable relationship between the patient and the therapist before, A more conservative attitude towards digital health interventions, Facilitate communication between mental health professionals and family physicians (Professional Interactions),Incentives and Resources (reasonable financial remuneration), Capacity for Organizational Change, - Patient factors: appropriate target groups for this type of intervention (Mobility of patients), The type of disorder as a factor for the use of this type of intervention, Some patients' concerns about video technology, |
| 18 | D. A. Lynch, et al. [18] | Client, clinician, and administrator factors associated with the successful acceptance of a telehealth comprehensive recovery service: A mixed methods study | 2021 | USA | To understand the multiple factors influencing the success of telehealth | A mixed method research (questionnaire & interview) | Telehealth | Patient (Client) and Provider (clinician and administrator) | Patient (client) and Provider (clinician and administrator) | People with SMI (serious mental illness) | - Factors from the point of view of administrator and clinician: Skepticism about telehealth, Client care challenges, Virtual etiquette, Managing group dynamics, Communication challenges, ZOOM fatigue, Staff perception of the telehealth conversion, Impact of organizational structure, Continuity of care, Clients’ familiarity with technology, Staff technological competency, Consistency of services, Staff responsiveness, Growing familiarity with technology platforms, Collaborative spirit in clients and staff, |
| 19 | K. J. W. Mendez, et al. [19] | Factors associated with intention to adopt m-health apps among dementia caregivers with a chronic condition: cross sectional, correlational study | 2021 | USA | To explore factors associated with dementia caregivers’ intention to adopt m-Health apps for chronic disease self-management | A mixed method research (telephone interview & web-based survey) | M-Health App | Provider (caregiver) | Patient and Provider (caregiver) | Dementia | - Perceived usefulness, Level of education, Burden of chronic disease (The probability of using m-Health programs among caregivers with a high level of education and burden of chronic disease and treatment was several times higher compared to people who had a low level of education and low burden of chronic diseases), |
| 20 | M. Michaelis, et al. [20] | Mental health applications for primary and secondary prevention of common mental disorders: attitudes of German employees | 2021 | Germany | Investigating of the attitudes of employees toward mental health apps and various traditional mental health services | Quantitative (survey) | Web based and mobile mental health applications | Patient (Employees) | Patient (Employee) | Common mental disorders (CMDs) | - The quality and effectiveness of the relevant programs, Literacy with electronic devices, Shame in the case of one’s own CMD, Willingness to begin a recommended psychotherapy in the case of one’s own CMD, Relevance of work demands for developing a CMD, |
| 21 | B. Naccache, et al. [21] | Smartphone application for adolescents with anorexia nervosa: an initial acceptability and user experience evaluation | 2021 | France | To explore early acceptability and user experience of a companion app prototype for adolescents with Anorexia nervosa | A mixed method research (questionnaire & group interview) | Smartphone application | Patient and Provider (practitioner) | Patient and Provider (practitioner) | Anorexia nervosa | - Installation barriers and facilitators: App’s design and graphics, Disorder induced phone disinterest, Shame, Denial, or disbelief in the app’s efficiency, Trusting the app’s data and believing in its usefulness, - Engagement barriers and facilitators: Adjustability of the apps, specifically in their activation, Notifications effect on disorder, Personalization features, App’s absence of testimonies, |
| 22 | J. Wang, et al. [22] | Preferred features of e-mental health programs for prevention of major depression in male workers: results from a Canadian national survey | 2016 | Canada | Examining the factors related to the use and possible barriers to the use of electronic mental health programs | Quantitative (survey) | E -Mental Health Programs | Patient (Male Workers) | Patient (Male Workers) | Depression | - Perceived usefulness, Features of e-mental health program, App`s format, Easiness to use, Confidentiality, Credibility, Direct link to a professional, Lack of personal interaction, Perceived stigma, |

**References:**

1. Austin, S.F., et al., *Service User Experiences of Integrating a Mobile Solution (IMPACHS) Into Clinical Treatment for Psychosis.* 2021. **31**(5): p. 942-954.

2. Bauer, M.S., et al., *Implementing and sustaining team-based telecare for bipolar disorder: lessons learned from a model-guided, mixed methods analysis.* 2018. **24**(1): p. 45-53.

3. Benjet, C., et al., *Treatment delivery preferences associated with type of mental disorder and perceived treatment barriers among Mexican university students.* 2020. **67**(2): p. 232-238.

4. Berry, N., F. Lobban, and S.J.B.p. Bucci, *A qualitative exploration of service user views about using digital health interventions for self-management in severe mental health problems.* 2019. **19**(1): p. 1-13.

5. Bleyel, C., et al., *Patients’ perspective on mental health specialist video consultations in primary care: qualitative preimplementation study of anticipated benefits and barriers.* 2020. **22**(4): p. e17330.

6. Chae, Y.M., et al., *The reliability and acceptability of telemedicine for patients with schizophrenia in Korea.* 2000. **6**(2): p. 83-90.

7. Chiu, T.M., G.J.B.m.i. Eysenbach, and d. making, *Stages of use: consideration, initiation, utilization, and outcomes of an internet-mediated intervention.* 2010. **10**(1): p. 1-11.

8. Coley, N., et al., *Older Adults' reasons for participating in an eHealth prevention trial: a cross-country, mixed-methods comparison.* 2019. **20**(7): p. 843-849. e5.

9. Cristancho-Lacroix, V., et al., *A web-based psychoeducational program for informal caregivers of patients with Alzheimer’s disease: a pilot randomized controlled trial.* 2015. **17**(5): p. e3717.

10. Bello-Haas, D.V., et al., *Lessons learned: feasibility and acceptability of a telehealth-delivered exercise intervention for rural-dwelling individuals with dementia and their caregivers.* 2014. **14**(3): p. [120]-[130].

11. Dias, L.P.S., et al., *Development and testing of iAware model for ubiquitous care of patients with symptoms of stress, anxiety and depression.* 2020. **187**: p. 105113.

12. Doyle, C., et al., *Videoconferencing and telementoring about dementia care: evaluation of a pilot model for sharing scarce old age psychiatry resources.* 2016. **28**(9): p. 1567-1574.

13. Fergie, G., et al., *Social media as a space for support: young adults' perspectives on producing and consuming user-generated content about diabetes and mental health.* 2016. **170**: p. 46-54.

14. Fitrianie, S., et al., *Factors Affecting User’s Behavioral Intention and Use of a Mobile-Phone-Delivered Cognitive Behavioral Therapy for Insomnia: A Small-Scale UTAUT Analysis.* 2021. **45**(12): p. 1-18.

15. Fonseca, A., R. Gorayeb, and M.C.J.I.j.o.m.i. Canavarro, *Women’s use of online resources and acceptance of e-mental health tools during the perinatal period.* 2016. **94**: p. 228-236.

16. Gately, M.E., et al., *Factors influencing barriers and facilitators to in-home video telehealth for dementia management.* 2021: p. 1-14.

17. Hoffmann, M., et al., *Perspectives of psychotherapists and psychiatrists on mental health care integration within primary care via video consultations: qualitative Preimplementation study.* 2020. **22**(6): p. e17569.

18. Lynch, D.A., et al., *Client, clinician, and administrator factors associated with the successful acceptance of a telehealth comprehensive recovery service: a mixed methods study.* 2021. **300**: p. 113871.

19. Mendez, K.J.W., et al., *Factors associated with intention to adopt mHealth apps among dementia caregivers with a chronic condition: cross-sectional, correlational study.* 2021. **9**(8): p. e27926.

20. Michaelis, M., et al., *Mental Health Applications for Primary and Secondary Prevention of Common Mental Disorders: Attitudes of German Employees.* 2021. **12**: p. 508622.

21. Naccache, B., et al., *Smartphone application for adolescents with anorexia nervosa: an initial acceptability and user experience evaluation.* 2021. **21**(1): p. 1-14.

22. Wang, J., et al., *Preferred features of e-mental health programs for prevention of major depression in male workers: results from a Canadian national survey.* 2016. **18**(6): p. e5685.
