# Supplementary material for: Factors affecting the acceptance of tele-psychiatry: a scoping study
Source: Arch Public Health. 2023 Jul 13;81:131. doi: 10.1186/s13690-023-01146-8 (PMC10339628; doi:10.1186/s13690-023-01146-8)
Supplement: Supplementary file 2 — Supplementary Material 2 [file 13690_2023_1146_MOESM2_ESM.docx]

| **Table B** Factors affecting the acceptance of telemedicine in the field of mental health | | | | |
| --- | --- | --- | --- | --- |
| **Reference** | **Final Codes** | **Sub-Factors** | **Main Factors** | **Item** |
| [1] | Valued recommendations | Denial of efficiency or belief in usefulness for improving health | Perceived effectiveness | 1 |
| [2] | Beliefs about treatment efficacy(positive) |  |  |  |
| [3], [4], [5] | Usefulness |  |  |  |
| [6] | Internet platform as an effective way to improve health, Benefiting from additional medical monitoring |  |  |  |
| [7] | Perceived usefulness for caregiver & patient |  |  |  |
| [8] | Performance expectancy |  |  |  |
| [9] | The perceived usefulness, the individual’s attitudes (effective) |  |  |  |
| [10], [11] | Perceived usefulness |  |  |  |
| [12] | The quality and effectiveness of the relevant programs |  |  |  |
| [13] | Trusting the app’s data and believing in its usefulness, Notifications effect on disorder |  |  |  |
| [14] | Digital health interventions as a positive way of self-management |  |  |  |
| [15] | Ability to focus without distraction due to telehealth |  |  |  |
| [16] | Supporting Memory (Reminder to complete treatment tasks) |  |  |  |
| [2] | Beliefs about treatment efficacy(negative) |  |  |  |
| [17] | Concerns about whether content is appropriate for sharing |  |  |  |
| [18] | Doubts about the appropriateness of video consultations to specialized mental health care |  |  |  |
| [13] | Denial, or disbelief in the app’s efficiency, |  |  |  |
| [7] | Preference of another service model from the patient's point of view (Because of not believing that anyone can help them.), |  |  |  |
| [19] | Skepticism about the usefulness of telehealth, |  |  |  |
| [14] | Digital health interventions as an attractive option to reduce the number of employees, reduce costs | Expected financial advantages of telemedicine |  |  |
| [20] | Reduce costs |  |  |  |
| [14] | Digital health interventions as a practical and interactive way of self-management, | Telemedicine as a tool for disease self-management | Effects of telemedicine on the quality and outcomes of care delivery | 2 |
| [20] | Relationship with other patients in the platform and get more general information about the disease, |  |  |  |
| [11] | Direct link to a professional, a way to strengthen self-sufficiency |  |  |  |
| [16] | Active follow-up of the disease as a result of promoting dialogue with a therapist |  |  |  |
| [21] | Increasing peer support and team building, | Telemedicine as a clinical decision support tool |  |  |
| [18] | Facilitate communication between mental health professionals and family physicians (Professional Interactions), |  |  |  |
| [12] | Relevance of work demands for developing a CMDs (Common mental disorders) |  |  |  |
| [6] | As an opportunity to interact | The effect of telemedicine on the quantity and quality of relationships |  |  |
| [3] | Quality of interpersonal relationship |  |  |  |
| [7] | Socialization |  |  |  |
| [18] | Time and duration of the individual video consultation (Face-to-face: 50 minutes, video consultation: 25 minutes; less time as a motivating factor) |  |  |  |
| [16] | Encouraging Reflection |  |  |  |
| [14] | Digital health interventions as powerful tools to stimulate reflection, understanding and change |  |  |  |
| [22] | Lack of Face-to-Face Contact as Anticipated Barrier, |  |  |  |
| [6] | The importance of social interaction and communication, |  |  |  |
| [18] | Time and duration of the individual video consultation (Face-to-face: 50 minutes, video consultation: 25 minutes; less time as a deterrent), The absence of personal interactions and non-verbal, |  |  |  |
| (18) | Communication challenges |  |  |  |
| [18] | Lack of personal interaction |  |  |  |
| [16] | Easily Accessible | Telemedicine as a pathway to easy access to services |  |  |
| [22] | Shorter Waiting Times, Shorter Travel Distances, Lower Threshold for Seeking Specialist Mental Health Care |  |  |  |
| [6] | Convenience of continuous access, |  |  |  |
| [20] | Increasing access to care, Potential to reduce the need for travel (both in terms of distance and traffic) |  |  |  |
| [7] | Flexibility, Dynamism |  |  |  |
| [15] | Ease of getting to the telehealth department |  |  |  |
| [21] | Reductions in travel times |  |  |  |
| [19] | Remote health sessions more convenient than in-person / increasing flexibility for better scheduling compliance, Maintaining continuity of care |  |  |  |
| [11] | Features of e-mental health program, App`s format, | Platform design based on aesthetic principles | Technological aspects | 3 |
| [13] | Adjustability of the apps, specifically in their activation, App’s design and graphics, |  |  |  |
| [19] | ZOOM fatigue |  |  |  |
| [7] | Personalization, | Personalization |  |  |
| [13] | Personalization features, |  |  |  |
| [8] | Feelings about app personalization, |  |  |  |
| [13] | App’s absence of testimonies, | Credibility |  |  |
| [11] | Credibility, |  |  |  |
| [8] | Trust, |  |  |  |
| [6] | sense of reliability and trustworthiness, |  |  |  |
| [14] | Concerns about who has access to DHI (Digital health interventions) data and how, | Privacy |  |  |
| [15] | How well privacy was respected, |  |  |  |
| [20] | Immunity of the platform in front of hackers, |  |  |  |
| [18] | A more conservative attitude towards digital health interventions |  |  |  |
| [11] | Confidentiality, |  |  |  |
| [6] | lack of conﬁdence in computer skills | Digital literacy and ease of learning and using telemedicine |  |  |
| [19] | Clients’ familiarity with technology, Staff technological competency, Growing familiarity with technology platforms, |  |  |  |
| [12] | Literacy with electronic devices |  |  |  |
| [10] | Level of education |  |  |  |
| [1] | Ease of use, Ease of use and integration into ongoing workflow via electronic health record for consulting providers |  |  |  |
| [4] | Caregivers' perception of ease of use |  |  |  |
| [5] | Easy to understand, Usability, |  |  |  |
| [8] | Effort expectancy |  |  |  |
| [9] | The perceived ease of use |  |  |  |
| [11] | Easiness to use |  |  |  |
| [20] | Ease of learning to use remote video health for ease of operation |  |  |  |
| [1] | Extensive infrastructure at the national level to support implementation, Inner (facility infrastructure) and outer (national policy and infrastructural support) context | Organizational-infrastructural challenges | Organizational change capacity | 4 |
| [18] | The challenge of providing incentives and resources (reasonable financial remuneration), |  |  |  |
| [8] | Facilitating conditions (The degree to which an individual believes an organizational and technical infrastructure exists to support the use of the system.) |  |  |  |
| [19] | Client care challenges |  |  |  |
| [21] | Internet speed |  |  |  |
| [14] | The digital divide |  |  |  |
| [22] | Technical Challenges |  |  |  |
| [22] | Possibility organizational challenges |  |  |  |
| [18] | Capacity for Organizational Change | Organizational structure and capabilities |  |  |
| [19] | Impact of organizational structure (organizational culture, organizational agility, resources and expertise for policy development), |  |  |  |
| [18] | Appropriate target groups for this type of intervention (Mobility of patients), The type of disorder as a factor for the use of this type of intervention, | Type of disorder | The nature of the disease, and psychological and psychosocial factors | 5 |
| [6] | The non-pharmacologic nature of the intervention |  |  |  |
| [19] | Consistency of services, |  |  |  |
| [10] | Burden of chronic disease (Mental disorder patients with chronic diseases have a greater tendency to use telemedicine than people without chronic diseases.) |  |  |  |
| [12] | Willingness to initiate recommended treatment in patients with CMD (Common mental disorders) |  |  |  |
| [2] | wanting to handle problems on one's own | Autonomy |  |  |
| [7] | Maintaining autonomy, |  |  |  |
| [8] | Self-efficacy |  |  |  |
| [2] | Being embarrassed | Embarrassment and fear of mental disorder stigma in society |  |  |
| [14] | Digital health interventions as an attractive option to reduce the fear of being judged in face-to-face visits |  |  |  |
| [22] | Stigma of Seeking Mental Health Care |  |  |  |
| [12] | Shame in the case of one’s own CMD |  |  |  |
| [13] | Shame |  |  |  |
| [11] | Perceived stigma |  |  |  |
| [8] | Anxiety |  |  |  |
| [14] | Digital health interventions as a fun way of self-management | Hedonic motivation |  |  |
| [6] | As a way to satisfy curiosity, Considering the internet as fun |  |  |  |
| [9] | The individual’s attitudes (attractive) |  |  |  |
| [8] | Hedonic motivation |  |  |  |
| [8] | Social influence | Social influence |  |  |
| [9] | The individual’s subjective norms (To encourage or not to encourage family and friends to use), The individual’s perceived behavioral (perceptions of internal (willingness to use) and external constraints (ability to use)) |  |  |  |
| [4] | Attitude towards higher technology | Desire for a technology-based lifestyle |  |  |
| [19] | Virtual etiquette, |  |  |  |
| [6] | Contributing to scientific progress and contributing to the development of an Internet health tool, wanting to improve one's lifestyle |  |  |  |
| [22] | Familiar Primary Care Environment | Feeling familiar due to previous experiences |  |  |
| [15] | Previous experiences |  |  |  |
| [22] | Familiar Primary Care Environment |  |  |  |
| [18] | The existence of a stable relationship between the patient and the therapist before |  |  |  |
| [3] | Feeling comfortable, Ease of self-expression | Ease of self- disclosure |  |  |
| [20] | Relative ease with video conferencing for personal reasons |  |  |  |

**References:**

1. Bauer, M.S., et al., *Implementing and sustaining team-based telecare for bipolar disorder: lessons learned from a model-guided, mixed methods analysis.* 2018. **24**(1): p. 45-53.

2. Benjet, C., et al., *Treatment delivery preferences associated with type of mental disorder and perceived treatment barriers among Mexican university students.* 2020. **67**(2): p. 232-238.

3. Chae, Y.M., et al., *The reliability and acceptability of telemedicine for patients with schizophrenia in Korea.* 2000. **6**(2): p. 83-90.

4. Chiu, T.M., G.J.B.m.i. Eysenbach, and d. making, *Stages of use: consideration, initiation, utilization, and outcomes of an internet-mediated intervention.* 2010. **10**(1): p. 1-11.

5. Dias, L.P.S., et al., *Development and testing of iAware model for ubiquitous care of patients with symptoms of stress, anxiety and depression.* 2020. **187**: p. 105113.

6. Coley, N., et al., *Older Adults' reasons for participating in an eHealth prevention trial: a cross-country, mixed-methods comparison.* 2019. **20**(7): p. 843-849. e5.

7. Cristancho-Lacroix, V., et al., *A web-based psychoeducational program for informal caregivers of patients with Alzheimer’s disease: a pilot randomized controlled trial.* 2015. **17**(5): p. e3717.

8. Fitrianie, S., et al., *Factors Affecting User’s Behavioral Intention and Use of a Mobile-Phone-Delivered Cognitive Behavioral Therapy for Insomnia: A Small-Scale UTAUT Analysis.* 2021. **45**(12): p. 1-18.

9. Fonseca, A., R. Gorayeb, and M.C.J.I.j.o.m.i. Canavarro, *Women’s use of online resources and acceptance of e-mental health tools during the perinatal period.* 2016. **94**: p. 228-236.

10. Mendez, K.J.W., et al., *Factors associated with intention to adopt mHealth apps among dementia caregivers with a chronic condition: cross-sectional, correlational study.* 2021. **9**(8): p. e27926.

11. Wang, J., et al., *Preferred features of e-mental health programs for prevention of major depression in male workers: results from a Canadian national survey.* 2016. **18**(6): p. e5685.

12. Michaelis, M., et al., *Mental Health Applications for Primary and Secondary Prevention of Common Mental Disorders: Attitudes of German Employees.* 2021. **12**: p. 508622.

13. Naccache, B., et al., *Smartphone application for adolescents with anorexia nervosa: an initial acceptability and user experience evaluation.* 2021. **21**(1): p. 1-14.

14. Berry, N., F. Lobban, and S.J.B.p. Bucci, *A qualitative exploration of service user views about using digital health interventions for self-management in severe mental health problems.* 2019. **19**(1): p. 1-13.

15. Bello-Haas, D.V., et al., *Lessons learned: feasibility and acceptability of a telehealth-delivered exercise intervention for rural-dwelling individuals with dementia and their caregivers.* 2014. **14**(3): p. [120]-[130].

16. Austin, S.F., et al., *Service User Experiences of Integrating a Mobile Solution (IMPACHS) Into Clinical Treatment for Psychosis.* 2021. **31**(5): p. 942-954.

17. Fergie, G., et al., *Social media as a space for support: young adults' perspectives on producing and consuming user-generated content about diabetes and mental health.* 2016. **170**: p. 46-54.

18. Hoffmann, M., et al., *Perspectives of psychotherapists and psychiatrists on mental health care integration within primary care via video consultations: qualitative Preimplementation study.* 2020. **22**(6): p. e17569.

19. Lynch, D.A., et al., *Client, clinician, and administrator factors associated with the successful acceptance of a telehealth comprehensive recovery service: a mixed methods study.* 2021. **300**: p. 113871.

20. Gately, M.E., et al., *Factors influencing barriers and facilitators to in-home video telehealth for dementia management.* 2021: p. 1-14.

21. Doyle, C., et al., *Videoconferencing and telementoring about dementia care: evaluation of a pilot model for sharing scarce old age psychiatry resources.* 2016. **28**(9): p. 1567-1574.

22. Bleyel, C., et al., *Patients’ perspective on mental health specialist video consultations in primary care: qualitative preimplementation study of anticipated benefits and barriers.* 2020. **22**(4): p. e17330.
